# A DWI-based hypoxia model shows robustness in an external prostatectomy cohort

**DOI:** 10.3389/fonc.2024.1433197

**Published:** 2024-07-23

**Authors:** M. Fernandez Salamanca, T. Hompland, M. Deręgowska-Cylke, H. Van der Poel, E. Bekers, M. A. S. Guimaraes, H. Lyng, U. A. Van der Heide, I. G. Schoots, P. J. Van Houdt

**Affiliations:** ^1^ Department of Radiology, The Netherlands Cancer Institute, Amsterdam, Netherlands; ^2^ Department of Radiation Biology, Oslo University Hospital, Oslo, Norway; ^3^ Department of Radiology, Medical University of Warsaw, Warsaw, Poland; ^4^ Department of Urology, The Netherlands Cancer Institute, Amsterdam, Netherlands; ^5^ Department of Urology, Amsterdam University Medical Centers, Amsterdam, Netherlands; ^6^ Department of Pathology, The Netherlands Cancer Institute, Amsterdam, Netherlands; ^7^ Department of Physics, University of Oslo, Oslo, Norway; ^8^ Department of Radiation Oncology, The Netherlands Cancer Institute, Amsterdam, Netherlands; ^9^ Department of Radiology & Nuclear Medicine, Erasmus University Medical Center, Rotterdam, Netherlands

**Keywords:** hypoxia, prostate cancer, diffusion weighted images, prostatectomy, hypoxia biomarker

## Abstract

**Introduction:**

Prostate cancer hypoxia is a negative prognostic biomarker. A promising MRI-based tool to assess hypoxia is the ‘Consumption and Supply based Hypoxia’ (CSH) model based on diffusion-weighted imaging (DWI). The aim of the study was to validate the association between the CSH hypoxia fraction (HF_DWI_) with pathological Grade Group (pGG) and pathological T-staging (pTstage) in an external prostatectomy cohort.

**Methods:**

Apparent diffusion coefficient (ADC) and fractional blood volume (fBV) maps were assessed from DWI data from 291 prostatectomies and combined by the CSH model. HF_DWI_ was calculated for each lesion after median scaling of ADC and fBV to address differences in acquisition and analysis between centers. The absolute HF_DWI_ values and the associations of HF_DWI_ between pGG < 3 versus ≥ 3, and pTstage = 2 versus = 3 in the Netherlands Cancer Institute (NKI) cohort were compared to the obtained by original cohort (Oslo cohort). Statistical T- and Mann-Whitney tests (*p*<0.05) were performed. Pearson correlation was determined between HF_DWI_ and individual pGG groups.

**Results:**

The HF_DWI_ showed comparable absolute values and similar metric performance as in the original published cohort. Higher HF_DWI_ values were observed for higher pGG (Oslo: 0.27; NKI: 0.24) compared to lower pGG (Oslo: 0.11; NKI: 0.17). Similar results were obtained for pTstage. Furthermore, HF_DWI_ demonstrated a significant positive correlation with pGG groups 1-5 (ρ = 0.41, *p*<0.001).

**Conclusion:**

The CSH model exhibited sufficient robustness in the external cohort, suggesting a plausible reflection of true hypoxia and enabling the use of the HF_DWI_ metric for further research into prostate cancer and hypoxia.

## 1 Introduction

Hypoxia in prostate cancer (PCa) has been related to radiation treatment resistance and metastatic disease (1–3). Thus, hypoxia assessment at diagnosis is of great interest for patient stratification and treatment decisions. Given that MRI is the main imaging modality in PCa patients and widely available in modern hospitals, the consideration of MRI biomarkers emerges as an attractive tool for treatment personalization. A promising approach, using diffusion-weighted images (DWI) related to oxygen consumption and supply, was developed by Hompland et al. (4). This model, known as Consumption and Supply based Hypoxia (CSH) imaging, relies on apparent diffusion coefficient (ADC) and fractional blood volume (fBV) maps and its application to PCa patients was based on the underlying assumption that the estimated ADC is linked to oxygen consumption while fBV is linked to oxygen supply.

The CSH model was trained using DWI data of PCa patients who received a hypoxia marker (pimonidazole) administration prior to prostatectomy as a ground truth to find the optimal combination of ADC and fBV representing hypoxia. In a separate test cohort, the hypoxia fraction (HF_DWI_) of the index lesion showed a robust correlation to hypoxia estimated from pimonidazole staining of the surgical specimen. Consequently, the CSH model appears as a promising non-invasive tool for hypoxia assessment, offering potential for personalized treatment decisions. Indeed this approach appeared to be quite successful for correlating the HF_DWI_ to pimonidazole-derived hypoxia in cancers such as breast and cervix (4–6).

To apply such a model more widely in clinical practice, external validation is necessary. A first hurdle is the potential variability in quantitative MRI parameters between scanners and centers (7, 8). Here we propose a calibration method by scaling the quantitative MRI parameters in matched cohorts of patients.

A second hurdle for validation of HF_DWI_ in relation to true hypoxia in prostate cancer is that it relies on the availability of pimonidazole staining. In the absence of pimonidazole stained specimen, we can make use of the established associations between hypoxia and pathological Grade Group (pGG), as well as Tstage (2, 4). Therefore, in this study we aim to compare the association between HF_DWI_ and pGG and pTstage in a cohort of patients who received a prostatectomy at the Netherlands Cancer Institute (NKI) and compare these associations with those originally obtained by Hompland et al (4).

## 2 Materials and methods

### 2.1 Cohort description

The original dataset from Oslo University Hospital consisted of 106 patients enrolled into the FuncProst study (NTC01464216) (4). For the external dataset, men with biopsy-proven prostate cancer and pre-operative MRI, who underwent radical prostatectomy between January 2010 and December 2020, were retrospectively included after approval of the institutional review board (IRBd21-108) at the NKI. Exclusion criteria were men with prior transurethral resection of the prostate, incomplete or technically poor-quality MRI or incomplete pathological specimens. A total of 291 patients were subjected to further analysis.

### 2.2 MRI acquisition and data analysis of the NKI cohort

MRI data in the NKI cohort were acquired using mostly a 3T scanner (Achieva [n=164], Achieva dStream [n=103] and Ingenia [n=21], Philips Healthcare, Best, the Netherlands). The MRI exam consisted of T2-weighted (T2w), DWI and a separate high b-value DWI acquisition (b= 1400 or 2000 s/mm^2^). Axial T2-weighted (T2w) turbo spin-echo images were acquired (Repetition time = [2175 – 10233 ms], Echo time = [110 – 130 ms]) with a field-of-view from 140 mm x 140 mm to 284 mm x 284 mm and slice thickness of 2.5 to 4 mm. DWI data were acquired with single-shot echo planar imaging sequences with b-values of 0, 200, 800 or 0,50,300,800 s/mm^2^. A detailed comparison of DWI acquisition parameters between centers can be found in **Supplementary Table S1**.

ADC and fBV maps were calculated using an intravoxel incoherent motion model with a segmented fit approach (9) ^1^. Details of this analysis can be found in **Supplementary Analysis**.

Tumor delineations on MR images (T2w, ADC and high b-value DWI scans) were performed by two observers (M.D.C. and M.F.S., both 1-2 years’ reading experience) blinded to the pathological ground truth. If required, consensus was reached after discussion with an experienced radiologist (I.G.S. 13 years of experience). Histological evaluation was performed by a pathologist (M.A.S.G., 10 years of experience) using SlideScore software [https://www.slidescore.com/]. Lesions larger than 3 mm were graded according to the 2019 International Society of Urological Pathology recommendations (10). Staging was performed according the TNM classification guidelines (11). For patients with multiple lesions, only the lesion with the highest pGG was used.

### 2.3 CSH model and calculation of hypoxia fraction

**Figure 1:**
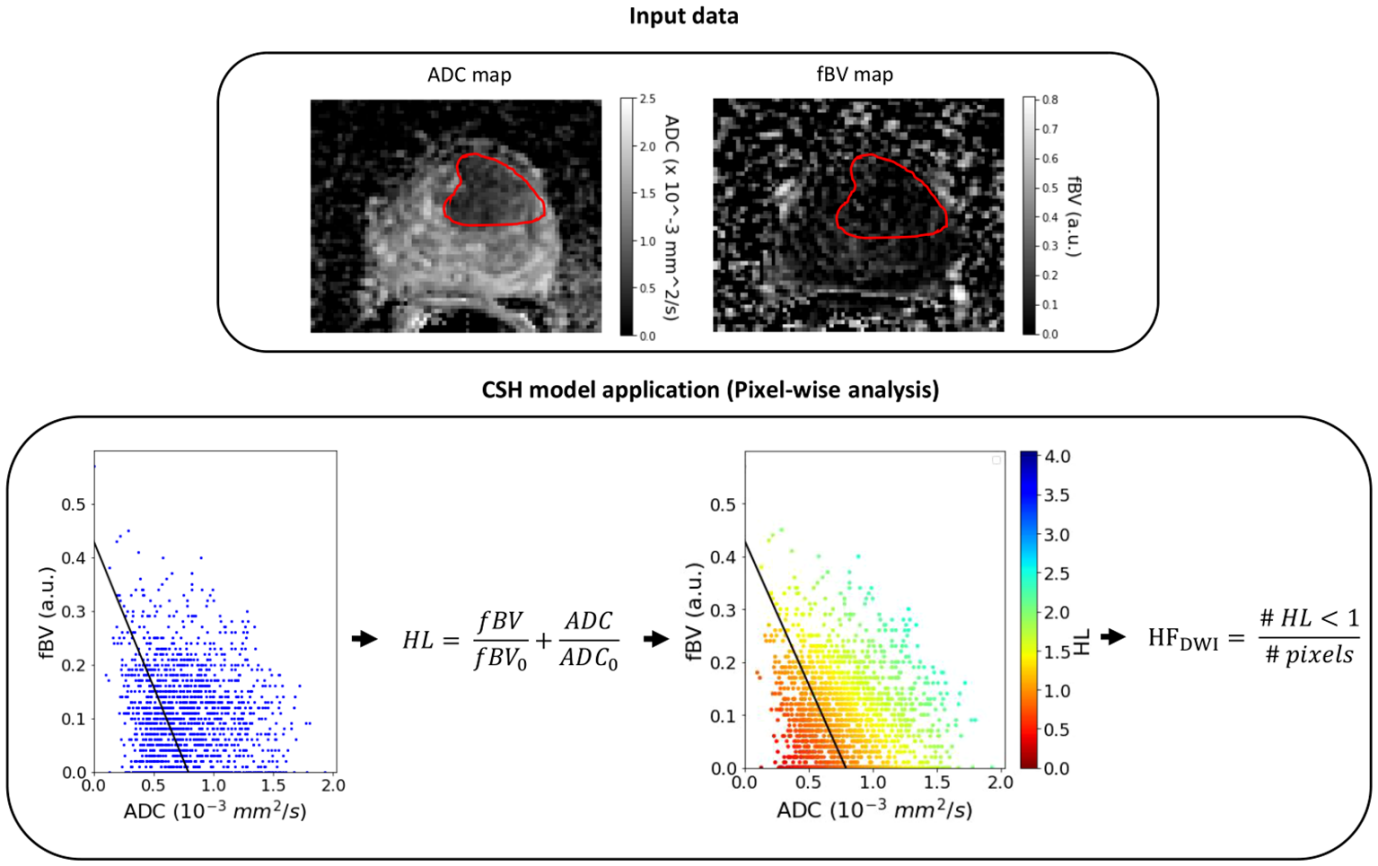
Example of the CSH model application on a lesion (red contour) of the NKI cohort. HL, hypoxia level (pixel-level); HF_DWI_, hypoxia fraction (lesion-level).

Previously, Hompland et al (4) showed that the discrimination of hypoxic and non-hypoxic regions could be approximated to a linear curve expressed as Equation (1)


(1) $$\frac{ADC}{ADC_{0}}+\frac{fBV}{fBV_{0}}=1$$

where ADC_0_ and fBV_0_ are the intersections with the ADC and fBV axes on a pixel-level plot (**Figure 1**). ADC_0_ = 0.79 x 10^-3^ mm^2^/s and fBV_0_ = 0.43 a.u. were the intercepts for which HF_DWI_ had the highest correlation with the hypoxia score from pimonidazole staining (HS_pimo_) in the Oslo cohort.

As differences in acquisition and analysis protocols between the two centers were present (**Supplementary Table S1**), specifically in the numbers of b-values used, median scaling of the ADC and fBV distributions was applied. However, to make sure that differences in clinical characteristics were not also affecting these variations, the cohorts were first matched based on clinical characteristics. For this purpose, propensity score matching was used and 106 patients from the NKI cohort were selected to clinically match the 106 patients dataset from the original institution (using pGG and pTstage as covariates). The decision to prioritize matching based on pGG and pTstage was driven by their known association with hypoxia (2, 4). When multiple lesions were present, the patient’s pGG corresponded to the lesion with the highest pGG. The R library MatchIt package (12) was used with *optimal* matching method, logistic regression distances and ‘Average Treatment Effect on the Control’ estimand (ATC), where only NKI patients are allowed to be dropped during the matching process, while maintaining Oslo number of patients fixed. This produced the best match based on standard mean differences.

For median scaling, the ADC and fBV voxel intensities distributions from all lesions of the matched NKI cohort were scaled towards the distributions of the Oslo dataset by multiplying them with the calculated scaled factors [F_ADC_ and F_fBV_, Equation (2)].


(2) $$\begin{array}{l} F_{ADC}=\frac{medianADC\left(Oslo\right)}{medianADC\left(NKI\right)}\\ F_{fBV}=\frac{medianfBV\left(Oslo\right)}{medianfBV\left(NKI\right)} \end{array}$$

Once the scaling was performed for the clinical matched set of patients, the HF_DWI_ metric was computed for each lesion using the ADC_0_ and fBV_0_ intercepts (**Figure 1**).

### 2.4 Hypoxia fraction validation and application

The absolute HF_DWI_ values were investigated by comparing the distributions within each pGG (low: pGG < 3 and high: pGG ≥ 3) and each pTstage group between centers. This comparison aimed to validate the consistency of HFDWI values between the two centers for each specific clinical subgroup.

Moreover, HF_DWI_ differences were examined between patients with low versus high pGG, and pTstage = 2 versus pTstage = 3 in the matched NKI cohort. These differences were then compared to the corresponding differences observed in the Oslo cohort. T-tests or Mann-Whitney tests (*p*< 0.05) were used for these comparisons, depending on normality and homogeneity (i.e. with equal variances) assessed by the Shapiro-Wilk test and Levene’s test, respectively.

Lastly, the CSH model was applied to the full NKI cohort (n=319 lesions from 291 patients) to investigate the HF_DWI_ relation and correlation across individual pGG groups using a Kruskal-Wallis (*p<* 0.05) and the Pearson correlation coefficient tests (ρ, *p<* 0.05). All analyses were performed using Python v3.7.

## 3 Results

The Oslo cohort was well balanced in terms of low and high pGG while overall dominated by pTstage 3 patients. In contrast, the full NKI cohort was dominated by low pGG and pTstage 2 patients. After propensity score matching, the matched NKI cohort had similar patient characteristics as the Oslo cohort (**Table 1**).

**Table 1:** Patient demographics and tumor characteristics for the original cohort (Oslo), the matched external cohort (NKI, pGG of the index lesion) and the full external cohort (Full NKI, with pGG information for all 319 lesions in 291 patients).

Finding	Oslo	NKI	Full NKI
**Patients’ external cohort**	n = 106	n = 106	n = 291
**Age (y)^a^**	65 (45 – 76)	66 (46 – 77)	67 (46 – 78)
**PSA (ng/ml)^a^**	8.9 (2 – 145)	8.6 (3 – 259)	8.3 (3 – 259)
Prostatectomy GG (pGG)			
**1**	8 (8)	8 (8)	45 (14)
**2**	44 (42)	44 (42)	147 (46)
**3**	26 (25)	26 (25)	76 (24)
**4**	16 (15)	16 (15)	30 (9)
**5**	12 (11)	12 (11)	21 (7)
Prostatectomy Tstage (pTstage)			
**2**	34 (32)	34 (32)	168 (58)
**3**	72 (68)	72 (68)	123 (42)

a median (min – max). GG, Grade Group; PSA, Prostate specific antigen.

With similar patient characteristics between the two cohorts we expect differences in ADC and fBV values to mostly reflect variations in the imaging acquisitions and MRI apparatus.

Prior to scaling, the matched NKI dataset showed slightly higher ADC values (median (IQR) was 0.84 (0.41) x10^-3^ mm/s^2)^ compared to the Oslo cohort (0.70 (0.26) x10^-3^ mm/s^2)^ and slightly lower fBV values (NKI: 0.08 (0.15) a.u. vs Oslo: 0.12 (0.11) a.u.) (**Supplementary Figure S1**). The obtained scaling factors *F*_ADC_ and *F*_fBV_ were 0.83 and 1.54, respectively.

**Figure 2:**
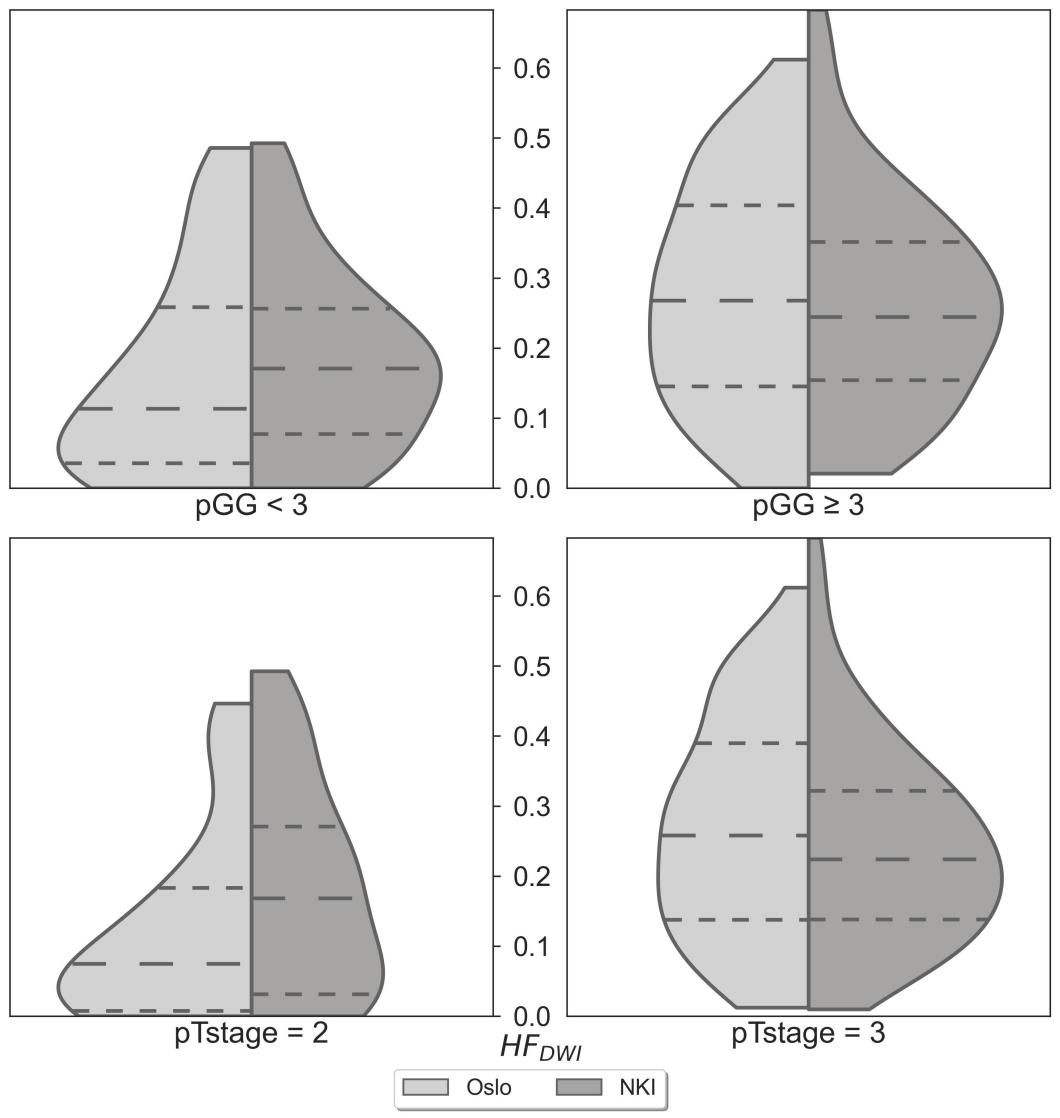
HF_DWI_ comparison between the Oslo (n=106) and NKI (n=106) datasets for specific pGG and pTstage sub-groups.

**Figure 3:**
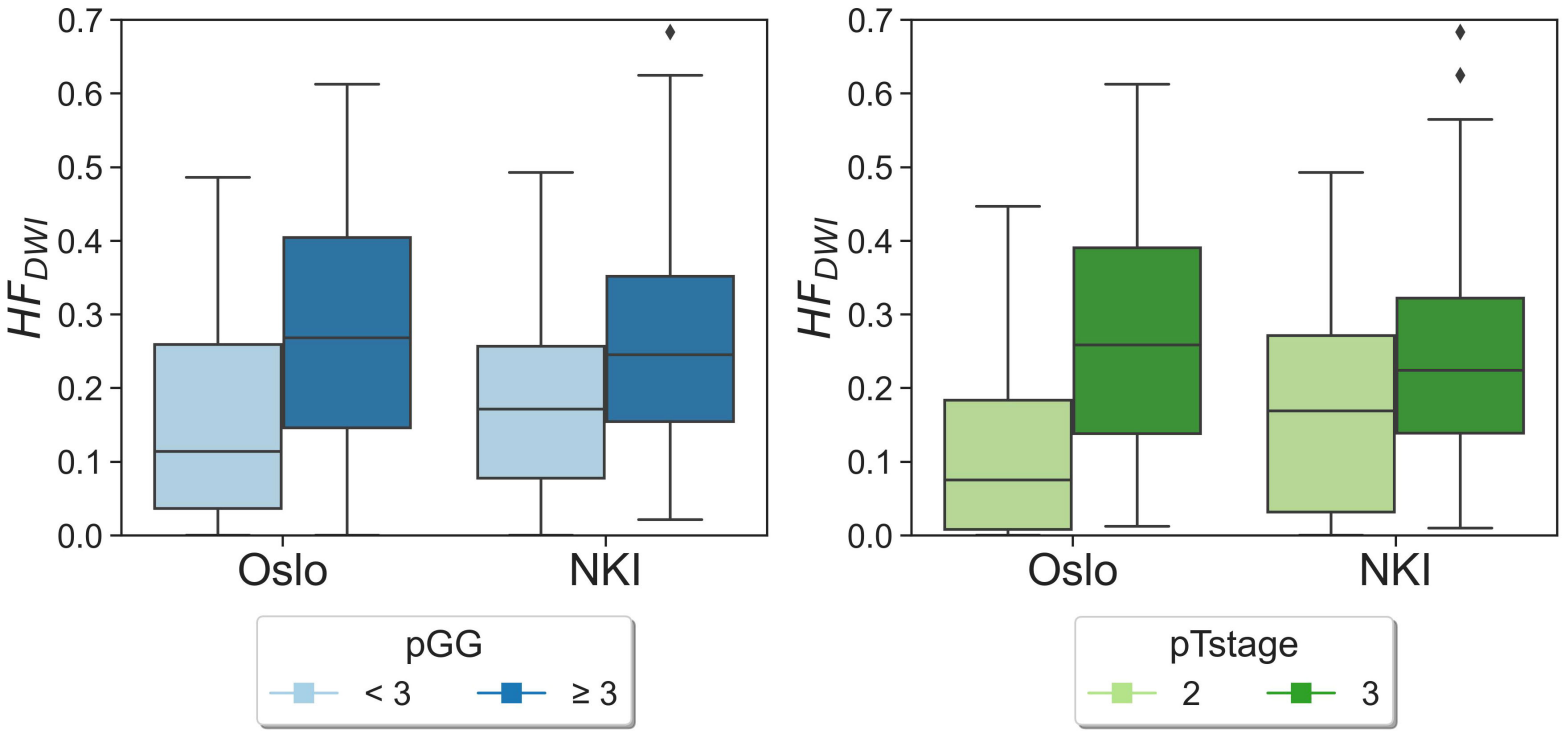
HF_DWI_ distributions for pGG and pTstage groups for both Oslo and NKI datasets. Diamond symbols represent outliers of the distributions.

**Figure 4:**
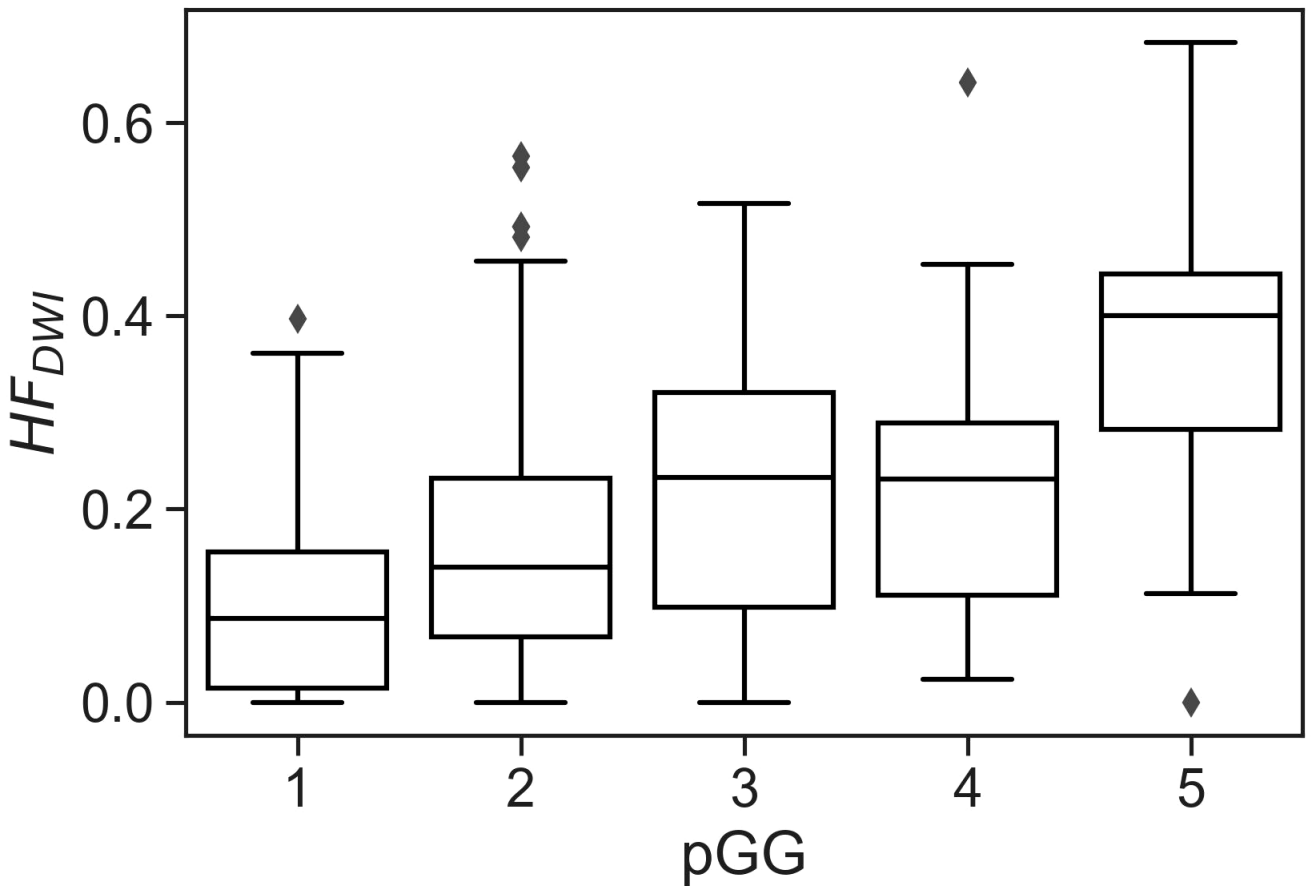
CSH model applied to the full NKI cohort (319 lesions from 291 patients) showing the correlation between hypoxia fraction with pGG individually. Kruskal-Wallis *p*< 0.001 indicates significant differences among groups.

No statistically significant differences were observed when comparing the matched NKI distributions of absolute HF_DWI_ values to the Oslo cohort for each specific pGG and pTstage subgroup (**Figure 2**, *p*(pGG<3) = 0.3, *p*(pGG>=3) = 0.8, *p*(pTstage=2) = 0.1, *p*(pTstage=3) = 0.4). HF_DWI_ median values for Oslo were 0.11 vs 0.27 for low and high pGG, and 0.08 vs 0.26 for pTstage = 2 and pTstage = 3. Median values for NKI were 0.17 vs 0.24 and 0.17 vs 0.22 for pGG and pTstage groups, respectively. The association between HF_DWI_ and pGG and pTstage was consistent between the Oslo and the matched NKI dataset (**Figure 3**): both showed that higher HF_DWI_ values were associated with higher pGG (Oslo p<0.001, NKI *p*=0.004) and pTstage groups (Oslo *p*<0.001, NKI *p*=0.03). Furthermore, significant differences were observed among all pGG groups in the full NKI cohort (**Figure 4**, *p<* 0.001), in addition to a significant positive correlation (ρ = 0.41, *p<* 0.001).

## 4 Discussion

The aim of this study was to independently validate the CSH model in prostate cancer by evaluating the HF_DWI_ association with pGG and pTstage in an independent external prostatectomy cohort.

After clinically matching and scaling, the HF_DWI_ in the NKI dataset exhibited similar associations with the pGG and pTstage groups as observed in the Oslo dataset. In agreement with Hompland et al. (4), a patient with a high HF_DWI_ in the NKI cohort was likely to exhibit more aggressive characteristics, such as a higher pTstage or pGG, in comparison to a patient with a lower HF_DWI_. Furthermore, the HF_DWI_ absolute values obtained in the NKI dataset were comparable to those of the Oslo dataset for both pGG and pTstage subgroups. While pimonidazole staining was unavailable in the NKI cohort, this similarity suggests that HFdwi may also indicate hypoxia in the NKI cohort.

Prior to scaling, the ADC and fBV distributions of the Oslo and NKI datasets were not very different (**Supplementary Figure S1**). This is an interesting observation as technical differences (including variations in vendor, sequence protocols, and data analysis methods) were present between the two cohorts. Nonetheless, for future applications of the CSH model in other datasets, ADC and fBV distributions need to be carefully compared with the Oslo data for an accurate use of the hypoxia metric. To allow other institutes to apply this method and determine scaling parameters for their cohort, all voxel values of ADC and fBV for the Oslo dataset and NKI matched dataset can be found in **Supplementary Data S1, Data S2** respectively. The framework for the CSH model application between different cohorts presented in this study offers a valuable template for transferring other quantitative MRI biomarkers between different cohorts.

As an example, the model was applied to the full available NKI cohort of 291 patients, showing consistent results for individual pGG categories. The positive correlation observed between HF_DWI_ and the individual pGG categories potentially positions the CSH-model as a non-invasive method to identify or classify patients into specific pGG groups.

A limitation of this study is the lack of pimonidazole staining to biologically validate the HF_DWI_ metric in an external cohort. Nevertheless, we showed that correlations between HF_DWI_ and measures of tumor aggressiveness and spread could be replicated in an external dataset.

MRI has the potential to non-invasively assess hypoxia in prostate cancer, shown by the current external validation of the relationship between HFDWI and pGG/pTstage, previously shown by Hompland et al. (4). MRI may thereby be capable in stratifying patients who are at a higher risk of worse clinical outcomes, e.g. disease progression or radiation resistance. Future research should focus on external validations across diverse clinical settings to ensure the robustness and generalizability of the CSH model.

## 5 Conclusion

The CSH model exhibited sufficient robustness in the external cohort, suggesting a plausible reflection of true hypoxia and enabling the use of the HF_DWI_ metric for further research into PCa and hypoxia.

## Data availability statement

The original contributions presented in the study are included in the article/**Supplementary Material**. Further inquiries can be directed to the corresponding author.

## Ethics statement

The studies involving humans were approved by the Netherlands Cancer Institute - IRBd21-108. The studies were conducted in accordance with the local legislation and institutional requirements. The ethics committee/institutional review board waived the requirement of written informed consent for participation from the participants or the participants’ legal guardians/next of kin because pursuant to national legislation prior to 25 May 2018 (Opt-out) and General hospital informed consent was considered.

## Author contributions

MF: Conceptualization, Data curation, Formal analysis, Investigation, Methodology, Software, Validation, Visualization, Writing – original draft, Writing – review & editing. TH: Conceptualization, Writing – review & editing, Software. M-DC: Data curation, Writing – review & editing. HV: Writing – review & editing. EB: Writing – review & editing. MG: Data curation, Writing – review & editing. HL: Writing – review & editing. UV: Supervision, Writing – review & editing. IS: Supervision, Writing – review & editing. PV: Conceptualization, Methodology, Supervision, Writing – review & editing.

## Footnotes

1 a https://github.com/OSIPI/TF2.4_IVIM-MRI_CodeCollection/tree/main/src/original/PvH_KB_NKI
