# Supplementary material for: A DWI-based hypoxia model shows robustness in an external prostatectomy cohort
Source: Front Oncol. 2024 Jul 23;14:1433197. doi: 10.3389/fonc.2024.1433197 (PMC11300287; doi:10.3389/fonc.2024.1433197)
Supplement: Supplementary file 1 [file DataSheet_1.docx]

Supplementary Material

**Table S1: Differences in DWI acquisition parameters between the Oslo and NKI cohorts.**

| Parameter | Oslo (n = 106) | Full NKI (n = 291) | |
| --- | --- | --- | --- |
| Magnetic field strength | 1.5T | 1.5T [n=3] | 3T [n=287] |
| Scanner | General Electric Discovery 450 magnet | Philips (Achieva [n=3]) | Philips (Achieva [n=161], Achieva dStream [n=103],Ingenia [n=21]) |
| Sequence | 2D SE-EPI | 2D SE-EPI | |
| FOV (mm^2^) | 180x180 | 381x381 | 180x180 to 256x256 |
| TR (ms) | 3000 | 4000 | 3466 to 6169 |
| TE (ms) | 59.4 | 70 | 54 to 104 |
| Echo train length | 92 | 83 to 87 | 55 to 113 |
| Slice Thickness (mm) | 4 | 3 to 3.3 | 2.73 to 4 |
| b-values (s/mm^2^) | 0,100,200,300,400,500,600,700,800,900,1000 | 0,200,800 or 0,50,300,800 | |

Note: Some information was missing for 10 of the NKI patients.

**Supplementary Analysis : DWI data analysis**

A simplified IVIM model (Equation 1) was used in both institutions under the assumption that D*>>ADC, using a b-value selection of 200 – 800 s/mm^2^ , where the signal loss is linear in a logarithmic plot(1).

| $\ln\left( \frac{S\left( b \right)}{S_{0}} \right)= -b\cdot ADC+\ln(1-fBV)$ | (1) |
| --- | --- |

In the NKI analysis, negative ADC and fBV voxel values were set to zero as they lacked physical significance. On the other hand, Hompland et al.(2) analysis allowed these negative values while building and testing the CSH model.

The differences in acquisition and analysis resulted in shape variations in ADC and fBV distributions between the two cohorts (Supplementary Figure 1). Especially for fBV the shape is different due to the difference in dealing with negative values. Nevertheless, the median scaling technique employed to compensate for technical differences (such as variations in MR protocols and scanner vendors) was robust to these differences in ADC and fBV analysis, as the median of the distributions remains unaffected.


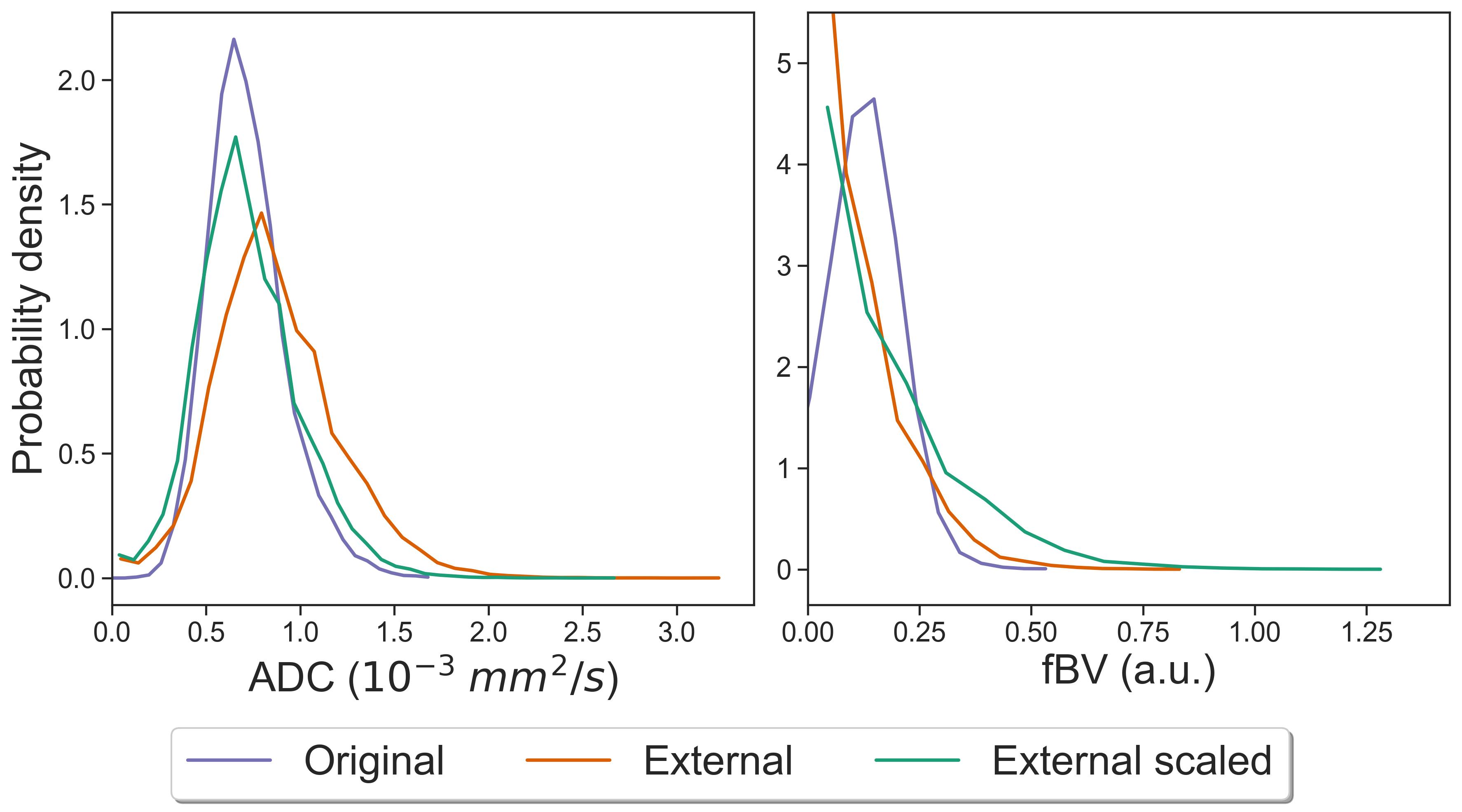


**Figure S1: ADC and fBV distributions for all tumors voxels for the Oslo cohort (n=106) and the NKI matched cohort (n=106) before and after median scaling.**

**References**

1. Kooreman ES, Van Houdt PJ, Keesman R, Van Pelt VWJ, Nowee ME, Pos F, et al. Daily Intravoxel Incoherent Motion (IVIM) In Prostate Cancer Patients During MR-Guided Radiotherapy—A Multicenter Study. Frontiers in Oncology. 2021;11.

2. Hompland T, Hole KH, Ragnum HB, Aarnes E-K, Vlatkovic L, Lie AK, et al. Combined MR Imaging of Oxygen Consumption and Supply Reveals Tumor Hypoxia and Aggressiveness in Prostate Cancer Patients. Cancer Research. 2018;78(16):4774-85.
